# From digital reminders to artificial intelligence–enabled understanding: can personalized treatment predictions improve therapeutic adherence?

**DOI:** 10.1093/ehjcvp/pvag079

**Published:** 2026-09-22

**Authors:** Valentin Goutaudier, Jean-Sébastien Hulot

**Affiliations:** PARCC, INSERM, Université Paris Cité, Paris F-75015, France; CIC1418 and DMU CARTE, AP-HP, Hôpital Européen Georges-Pompidou, Paris F-75015, France; PARCC, INSERM, Université Paris Cité, Paris F-75015, France; CIC1418 and DMU CARTE, AP-HP, Hôpital Européen Georges-Pompidou, Paris F-75015, France

## Abstract

**Graphical Abstract:**
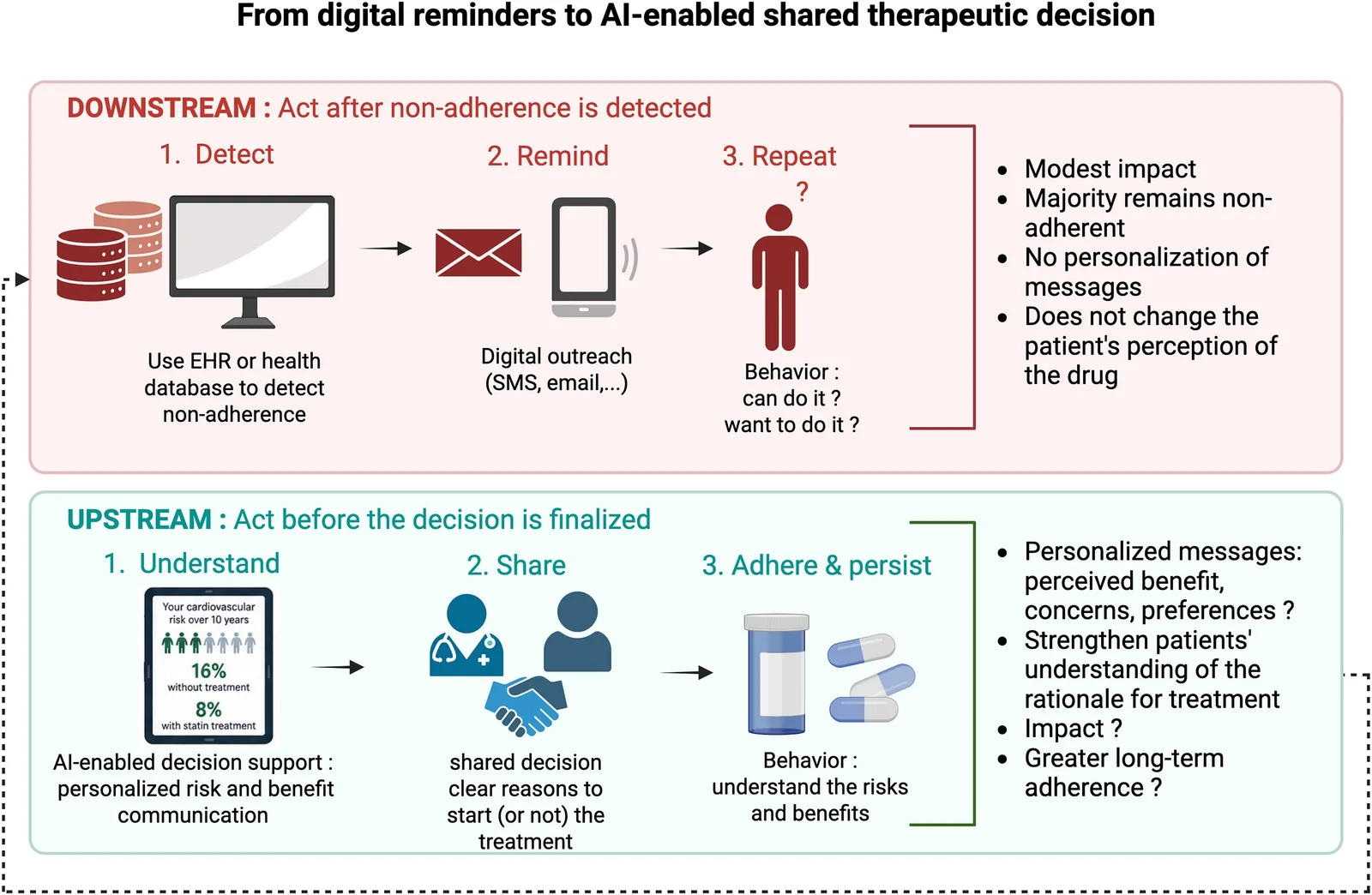

The effectiveness of cardiovascular prevention depends not only on prescribing evidence-based therapies, but also on patients taking them. Yet, poor adherence to long-term cardiovascular treatment remains remarkably common. Statins provide a striking example: real-world studies indicate that approximately one-third to one-half of patients are inadequately adherent or discontinue treatment over time, despite well-established cardiovascular benefits.^1–3^

Digital health appears to offer an attractive solution. Pharmacy dispensing or refill data can identify patients with potential non-adherence at scale, while patient portals, text messages, and emails provide inexpensive means of reaching them. Artificial intelligence (AI) further raises the prospect of predicting non-adherence and delivering personalized interventions. But a simpler question remains: are we intervening at the right point in the adherence pathway?

The recently presented ADHERE-ASCVD trial^4^ randomized 20 604 adults with atherosclerotic cardiovascular disease (ASCVD) or type 2 diabetes and recent statin non-adherence to patient-portal messaging, SMS/text messaging, email, or usual communication. Digital outreach significantly increased statin refill within 14 days from 12.0% to 14.4%, while repeated outreach or switching communication modality provided little additional benefit.^4^ At population level, even a small absolute improvement from a low-cost intervention may translate into meaningful numbers of patients restarting therapy. However, the finding that more than 85% of patients still did not refill highlights a clear ceiling of reminder-based strategies.

## Non-adherence is not simply a failure to remember

Medication non-adherence is often approached as a behavioural problem that reminders can correct. Non-adherence is heterogeneous and reflects patient-, treatment-, healthcare-system-, and socioeconomic factors.^1,5^ At the risk of oversimplification, patients may include those who cannot take treatment because of cost, access, complexity, or adverse effects, and those who choose not to because its expected benefit does not justify its perceived burden. Neither situation is necessarily resolved by another reminder.

This may be particularly important in cardiovascular prevention. Treatment is taken today to prevent an event that may occur years later while perceived adverse effects and medication burden are immediate. Perceptions of medication necessity and concerns are recognized determinants of adherence.^5,6^ Communicating the balance between an abstract future benefit and a tangible present burden is therefore intrinsically difficult.

Recent data from the QregPV cohort^7^ illustrate how therapeutic adherence may vary with clinical context. Adequate statin adherence was 54.0% in primary prevention, 61.3% among patients with diabetes without cardiovascular disease, and 65.5% in secondary prevention. Although these observational data cannot explain why adherence differed, they raise an important hypothesis: patients may be more likely to persist with preventive therapy when its personal relevance and expected benefit are more apparent. Variation across primary healthcare centres further suggests that how and where treatment is proposed may also matter.^7^

## Perhaps the AI-based intervention should start before non-adherence

Many digital adherence strategies intervene downstream: non-adherence is detected, and the patient is contacted. Systematic reviews suggest that digital interventions can improve medication adherence, but with heterogeneous and often modest effects.^4,8,9^ What if greater effort were directed upstream, when patients first decide whether the treatment makes sense to them?

For clinicians, a statin indication may appear self-evident from LDL-cholesterol, previous ASCVD, or high cardiovascular risk estimation. For patients, the question may be simpler: ‘What will this treatment actually do for me?’. Answering it well is difficult, particularly during a conventional consultation. Absolute benefit depends on baseline risk and treatment duration, while numeracy, health literacy, language, cultural background, previous experience, and preferences differ substantially. How risk information is presented itself influences understanding.^10,11^ A standardized explanation is therefore unlikely to be equally meaningful to every patient.

Digital tools, particularly AI-enabled decision support systems, could have a more ambitious role than delivering reminders. Initially developed to support clinicians in therapeutic decision-making, these tools could also support patients in collectively deciding whether to initiate and persist with a treatment. By integrating validated risk models and treatment effects, they could translate individualized benefits and harms into absolute risk reduction, cardiovascular events prevented, event-free survival, or graphical representations of treated and untreated risk, adapted to language and health literacy. Rather than returning to a paternalistic model or simply providing patients with neutral information, AI-enabled tools could support a more collaborative form of decision-making, in which personalized predictions make the expected benefit of treatment more tangible and personally meaningful. By strengthening patients’ understanding of the rationale for treatment, such tools may ultimately increase their willingness to initiate and persist with therapy.

This concept is not new. The Statin Choice decision aid showed nearly two decades ago that individualized presentation of cardiovascular risk and statin benefit could improve knowledge and aspects of shared decision-making.^12^ However, this small trial preceded contemporary digital and AI-enabled decision support. More recently, the Initial Medication Adherence intervetion cluster Randomized Controlled Trial (IMA-cRCT) study tested a pragmatic primary-care intervention incorporating shared decision-making and treatment decision aids at initiation of cardiovascular and diabetes therapies.^13^ Yet evidence that personalized risk communication translates into sustained long-term medication adherence remains limited. This is precisely the gap future randomized trials should address.

Importantly, this is not an argument for delegating treatment decisions to an algorithm or chatbot. AI should support both clinician and patient by translating evidence into individualized, understandable predictions, not automate persuasion. A patient who understands the expected benefits and harms but decides against treatment presents a fundamentally different situation from one who leaves the consultation without understanding why long-term treatment was recommended.

## From prediction to understanding

Much current enthusiasm around AI and adherence focuses on predicting who will become non-adherent. Such prediction may improve targeting,^6,7^ but AI could also act upstream, translating treatment-benefit predictions into patient-facing decision support rather than another generic reminder.

The next generation of digital adherence studies should therefore investigate not only who to contact and through which channel, but what should be communicated, how it should be personalized, and when. Randomized trials should determine whether AI-enabled decision support systems at treatment initiation improve informed treatment acceptance, long-term adherence, and ultimately clinical outcomes.

ADHERE-ASCVD demonstrates that large-scale digital outreach can move the needle on medication refill. Its modest absolute effect should encourage us to reconsider where digital approaches are deployed. AI-embedded digital systems may achieve more if it moves upstream: translating cardiovascular evidence into personalized predictions that are meaningful when the treatment decision is being made (Central illustration).

The challenge may be less about reminders than about using digital tools and AI to give each patient a clear, personalized reason and confidence to adhere and persist.

## Data Availability

No new data were generated or analysed in support of this research.
